# Intracranial Rosai–Dorfman Disease Presenting as a Posterior Fossa Dural-Based Mass Mimicking Meningioma: A Case Report

**DOI:** 10.3390/diagnostics16203384

**Published:** 2026-10-18

**Authors:** Ahmed Najjar, Zeyad Al-Shaibani, Nusaibah Basahel, Obai M. Raffah, Yasir Hassan Elhassan

**Affiliations:** 1 Department of General and Specialized Surgery, College of Medicine, Taibah University, Madinah 42353, Saudi Arabia; anajjar@taibahu.edu.sa; 2 Department of Surgery, King Faisal Specialist Hospital and Research Center, Madinah 42353, Saudi Arabia; 3 Department of Hematology, King Faisal Specialist Hospital and Research Center, Madinah 42353, Saudi Arabia; zeyadalshaibani@kfshrc.edu.sa; 4 Department of Pathology, King Faisal Specialist Hospital and Research Center, Madinah 42353, Saudi Arabia; nbasahel@kfshrc.edu.sa; 5 College of Medicine, Al-Rayan Medical Colleges, Madinah 42353, Saudi Arabia; obairaffah@gmail.com; 6 Department of Basic Medical Sciences, College of Medicine, Taibah University, Madinah 42353, Saudi Arabia

**Keywords:** Rosai–Dorfman disease, non-Langerhans cell histiocytosis, intracranial lesion, posterior fossa, dural-based mass, meningioma mimic, emperipolesis, case report

## Abstract

**Background and Clinical Significance:** Rosai–Dorfman disease (RDD) is a rare non-Langerhans-cell histiocytosis that infrequently involves the central nervous system. Intracranial RDD commonly presents as a dural-based enhancing mass that radiologically resembles a meningioma. Posterior fossa involvement is particularly uncommon and may cause cerebellar compression and obstructive hydrocephalus. **Case Presentation:** A 58-year-old man with biopsy-confirmed systemic extranodal RDD presented with progressive headache, nausea, severe dizziness, gait instability, cerebellar ataxia, and right upper-limb dysmetria despite corticosteroid therapy. Brain magnetic resonance imaging demonstrated a strongly enhancing right posterior fossa dural-based mass with vasogenic edema, cerebellar compression, fourth-ventricular effacement, and mild obstructive hydrocephalus. A smaller anterior dural-based enhancing lesion was also identified. The radiological differential diagnosis included meningioma, metastatic disease, and an inflammatory lesion. Following multidisciplinary perioperative management of anemia and complex transfusion incompatibility, gross-total resection of the symptomatic posterior fossa lesion was achieved. Histopathological examination demonstrated histiocytic proliferation with emperipolesis. The lesional histiocytes expressed S100 and CD68, showed nuclear Cyclin D1 expression in approximately 30% of lesional histiocytes, and were negative for CD1a, supporting the diagnosis of intracranial extranodal RDD. Postoperatively, the patient’s headache and cerebellar manifestations improved. Follow-up imaging demonstrated complete resection of the posterior fossa lesion, resolution of the mass effect and hydrocephalus, and no recurrence at the resection site. Extracranial disease subsequently progressed, emphasizing the distinction between local and systemic disease control. **Conclusions:** Intracranial RDD should be considered in the differential diagnosis of meningioma-like posterior fossa masses, particularly in patients with systemic RDD. Histopathological and immunohistochemical confirmation is essential. Timely surgical resection can provide a definitive diagnosis, effective decompression, and sustained neurological improvement, while continued systemic surveillance remains necessary.

## 1. Introduction

Rosai–Dorfman disease (RDD), also known as Rosai–Dorfman–Destombes disease, is a rare non-Langerhans-cell histiocytosis characterized by the accumulation of activated histiocytes within nodal or extranodal tissues. Its classic presentation consists of painless, massive cervical lymphadenopathy, although extranodal involvement occurs in approximately 40% of patients and may affect the skin, upper respiratory tract, bone, soft tissues, and central nervous system (CNS) [1]. Histologically, RDD is characterized by large histiocytes exhibiting emperipolesis. The characteristic immunophenotype generally includes expression of S100, CD68, and CD163, with absent CD1a expression [1,2]. Although CD163 is a recognized marker of RDD, it was not tested in the present case.

CNS involvement is uncommon, occurring in fewer than 5% of reported RDD cases [1]. Intracranial disease usually manifests as one or more strongly enhancing, dural-based masses that resemble meningiomas on computed tomography (CT) and magnetic resonance imaging (MRI) [3,4]. Consequently, imaging alone is generally insufficient to establish the diagnosis. Other radiological differential diagnoses include lymphoma, metastatic disease, inflammatory pseudotumor, and solitary fibrous tumor [3,4]. Histopathological examination and immunohistochemical characterization therefore remain essential for definitive diagnosis.

Posterior fossa involvement is particularly uncommon but may have serious clinical consequences because of the restricted anatomical space and proximity to the cerebellum, brainstem, and fourth ventricle [5]. Lesions in this location may cause cerebellar dysfunction, brainstem compression, obstructive hydrocephalus, and progressive intracranial hypertension. Surgical resection may therefore serve both diagnostic and therapeutic purposes when a lesion causes neurological deficits or clinically significant mass effect [1].

We report a patient with biopsy-confirmed systemic extranodal RDD who developed a symptomatic, dural-based posterior fossa mass radiologically resembling a meningioma. The lesion caused cerebellar compression, fourth-ventricular effacement, and mild obstructive hydrocephalus and was treated with gross-total resection. This report highlights the diagnostic value of radiology–pathology correlation, the role of surgery in symptomatic intracranial RDD, and the importance of considering RDD when evaluating a meningioma-like mass in a patient with known systemic disease. This case report was prepared in accordance with the CAse Report (CARE) guidelines. The completed CARE checklist is provided in Supplementary Table S1.

## 2. Case Presentation

A 58-year-old man with diabetes mellitus and a solitary kidney was initially evaluated for iron-deficiency anemia and gastrointestinal bleeding. Upper gastrointestinal endoscopy revealed gastritis without other gastrointestinal lesions. The patient had multiple subcutaneous nodules that were initially suspected clinically to represent neurofibromatosis. A representative chest-wall nodule was biopsied and demonstrated extranodal Rosai–Dorfman disease (RDD). The remaining subcutaneous nodules were not biopsied; therefore, their histopathological nature could not be definitively established as either RDD lesions or neurofibromas. Molecular testing showed no detectable BRAF V600E mutation. The patient also reported an unintentional weight loss of approximately 30 kg over six months.

A posterior fossa mass had previously been identified during an evaluation for headache, but the patient initially declined surgical treatment. For systemic RDD, the patient initially received oral prednisone at a dose of 20 mg once daily for two months. This produced a limited radiological response and was associated with hyperglycemia, which was treated and monitored by the endocrinology team. Prednisone was subsequently replaced with oral dexamethasone at a dose of 4 mg once daily. Dexamethasone continued for four months, from approximately two months after diagnosis until surgery, during which the disease remained radiologically stable. Dexamethasone was tapered during the perioperative period with hydrocortisone coverage under endocrinological supervision. No other major dexamethasone-related complications were documented. The patient was referred to the neurosurgical service because of worsening headache, nausea, severe dizziness, and gait instability.

On examination, the patient was conscious, oriented, and hemodynamically stable. Neurological assessment demonstrated cerebellar ataxia and right upper limb dysmetria. No cranial nerve deficits were detected, and motor strength and sensory function were preserved.

**Figure 1:**
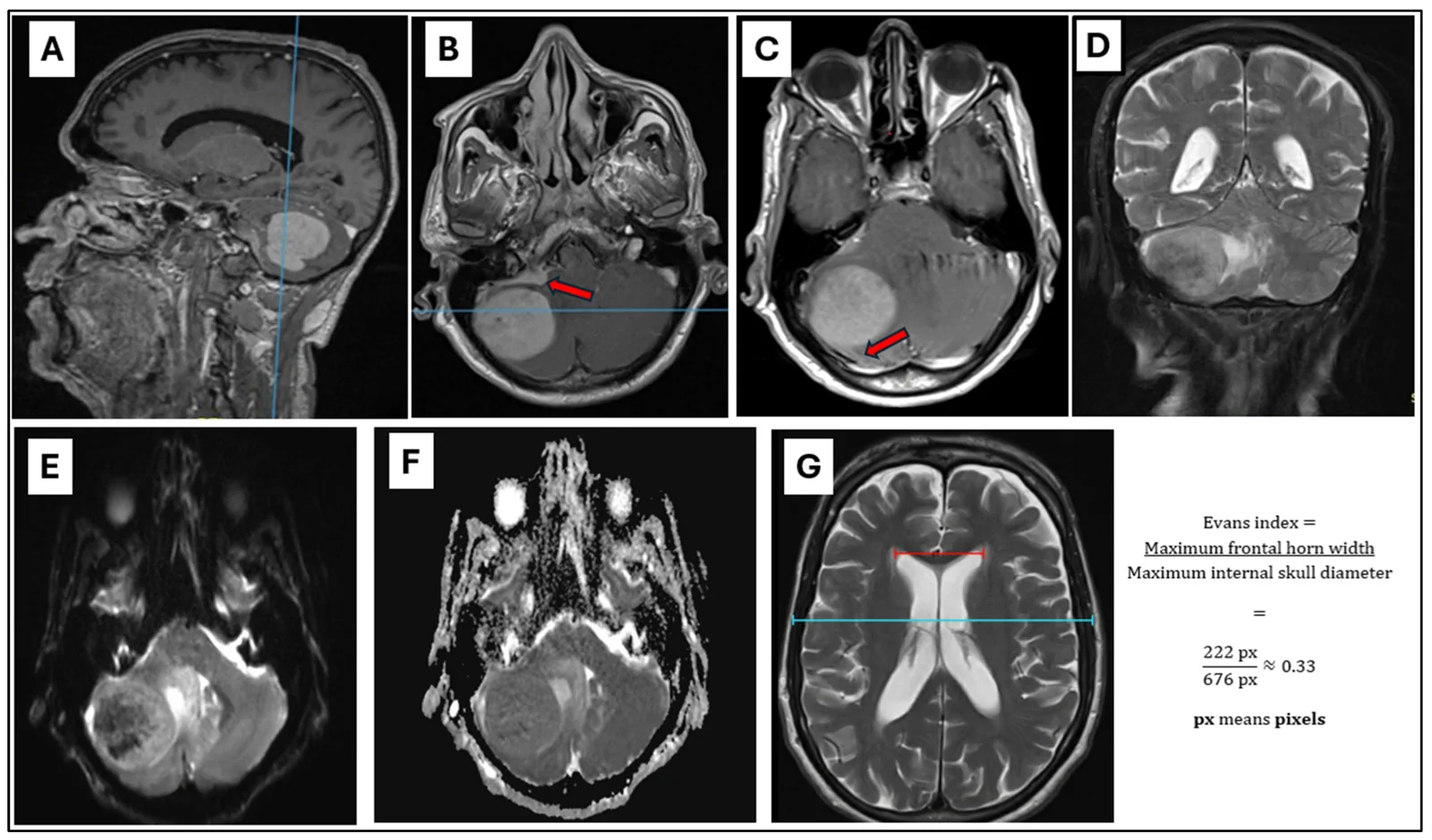
Preoperative brain magnetic resonance imaging. (**A**) Sagittal and (**B**,**C**) axial gadolinium-enhanced T1-weighted images demonstrate a large, strongly enhancing, dural-based right posterior fossa mass with substantial cerebellar mass effect. The arrow in (**B**) indicates a smaller anterior dural-based enhancing lesion, and the arrow in (**C**) indicates the dural attachment/tail of the dominant posterior fossa lesion. (**D**) Coronal T2-weighted image demonstrates surrounding vasogenic edema, cerebellar compression, and effacement of the fourth ventricle. (**E**) Diffusion-weighted image and (**F**) corresponding apparent diffusion coefficient map demonstrate the posterior fossa lesion. (**G**) Axial T2-weighted image demonstrates enlargement of the lateral ventricles, consistent with mild obstructive hydrocephalus. The Evans index, calculated as the maximum frontal horn width divided by the maximum internal skull diameter at the same axial level, is approximately 0.33.

Brain computed tomography showed a posterior fossa mass causing cerebellar compression and effacement of the fourth ventricle (Figure 1D), with mild obstructive hydrocephalus (Figure 1G). Magnetic resonance imaging revealed a well-defined, strongly enhancing, dural-based mass in the right posterior fossa (Figure 1A–C). The lesion exerted substantial mass effect on the cerebellum and fourth ventricle and was associated with surrounding vasogenic edema. A smaller anterior dural-based enhancing lesion was also present (Figure 1B). The principal radiological differential diagnoses included meningioma, metastatic disease, and an inflammatory lesion. Although the patient had known systemic RDD, imaging alone could not reliably distinguish intracranial RDD from these entities. No preoperative cerebrospinal fluid diversion procedure, including external ventricular drainage or endoscopic third ventriculostomy, was performed. The mild obstructive hydrocephalus was managed by resection of the posterior fossa lesion responsible for fourth-ventricular compression.

Because of the progressive cerebellar manifestations, compression of the fourth ventricle (Figure 1D), and need for a definitive tissue diagnosis, surgical resection was recommended after multidisciplinary consultation involving the neurosurgery, hematology, radiology, pathology, anesthesiology, and transfusion medicine teams.

Preoperative management was complicated by anemia and serological incompatibility during crossmatching. Extended compatibility testing failed to identify a fully compatible donor unit. After consultation with the transfusion medicine service, a least-incompatible, irradiated, leukoreduced O-positive packed red blood cell unit was selected and transfused under close clinical monitoring, including serial assessment of vital signs. No acute transfusion reaction occurred. Surgery proceeded after suitable blood products had been made available for potential intraoperative use.

The patient underwent microsurgical resection of the posterior fossa lesion through a suboccipital approach in the prone position. Intraoperatively, the dura was found to be infiltrated by the lesion and was excised with the involved tumor. The tumor was highly vascular and was removed in a piecemeal fashion. Bleeding arose from the tumoral vessels and was controlled using bipolar electrocautery and topical hemostatic materials, including Surgicel and TachoSil. Complete hemostasis was achieved without intraoperative complications. The estimated blood loss was approximately 500 mL, and one unit of packed red blood cells was transfused without complication. Gross-total resection was achieved. No external ventricular drain, endoscopic third ventriculostomy, or other separate procedure for hydrocephalus was performed because removal of the obstructing posterior fossa lesion restored cerebrospinal fluid flow.

**Figure 2:**
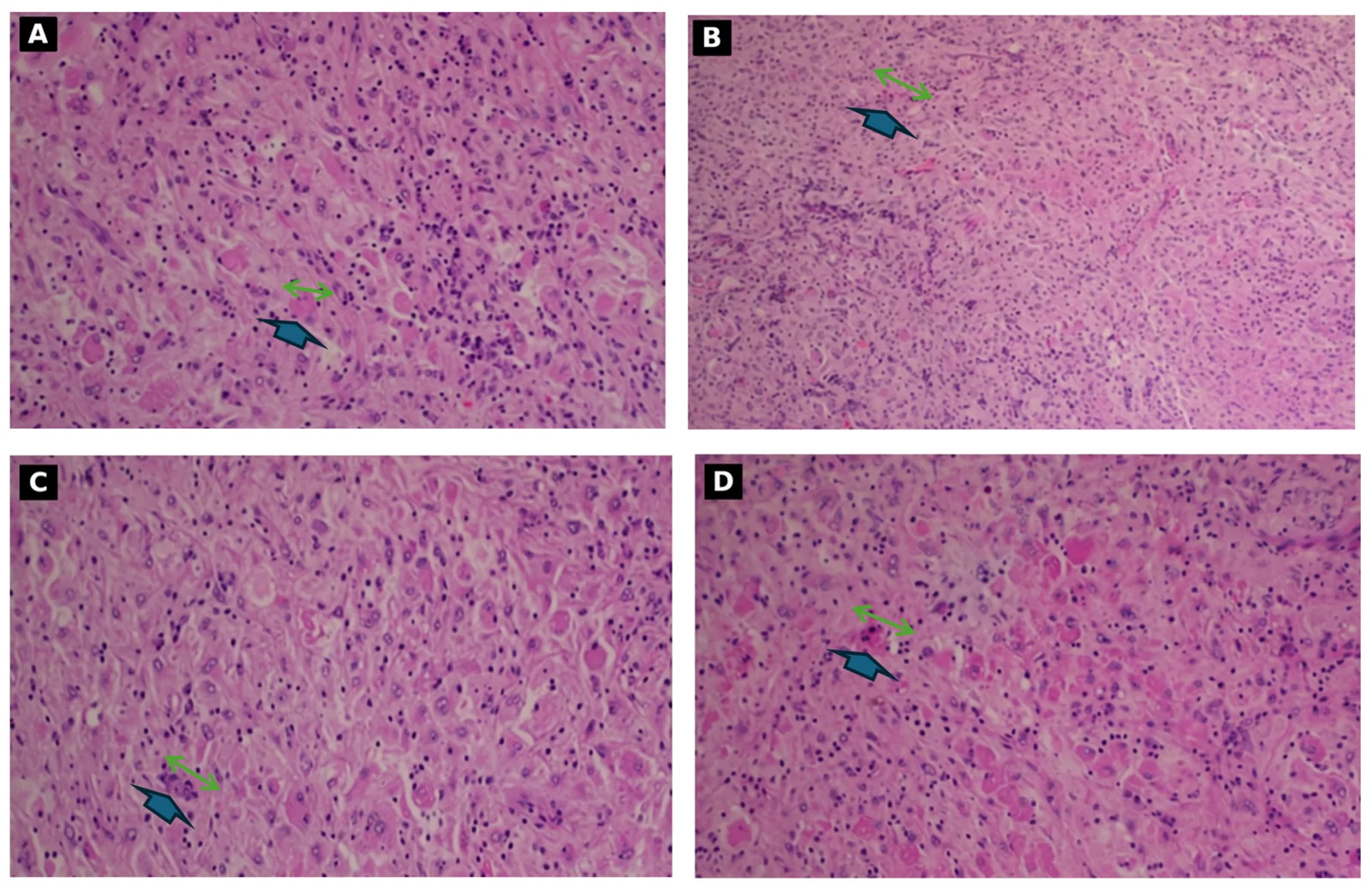
Histopathological features of the posterior fossa lesion. Representative hematoxylin and eosin (H&E)-stained sections demonstrate large, pale lesional histiocytes admixed with numerous lymphocytes and plasma cells in a mildly fibrotic background. Blue arrows indicate representative foci of emperipolesis, characterized by intact inflammatory cells within the cytoplasm of lesional histiocytes. Green lines indicate the extent of the highlighted histological areas. Original objective magnifications: (**A**) ×20, (**B**) ×10, (**C**) ×20, and (**D**) ×20.

**Figure 3:**
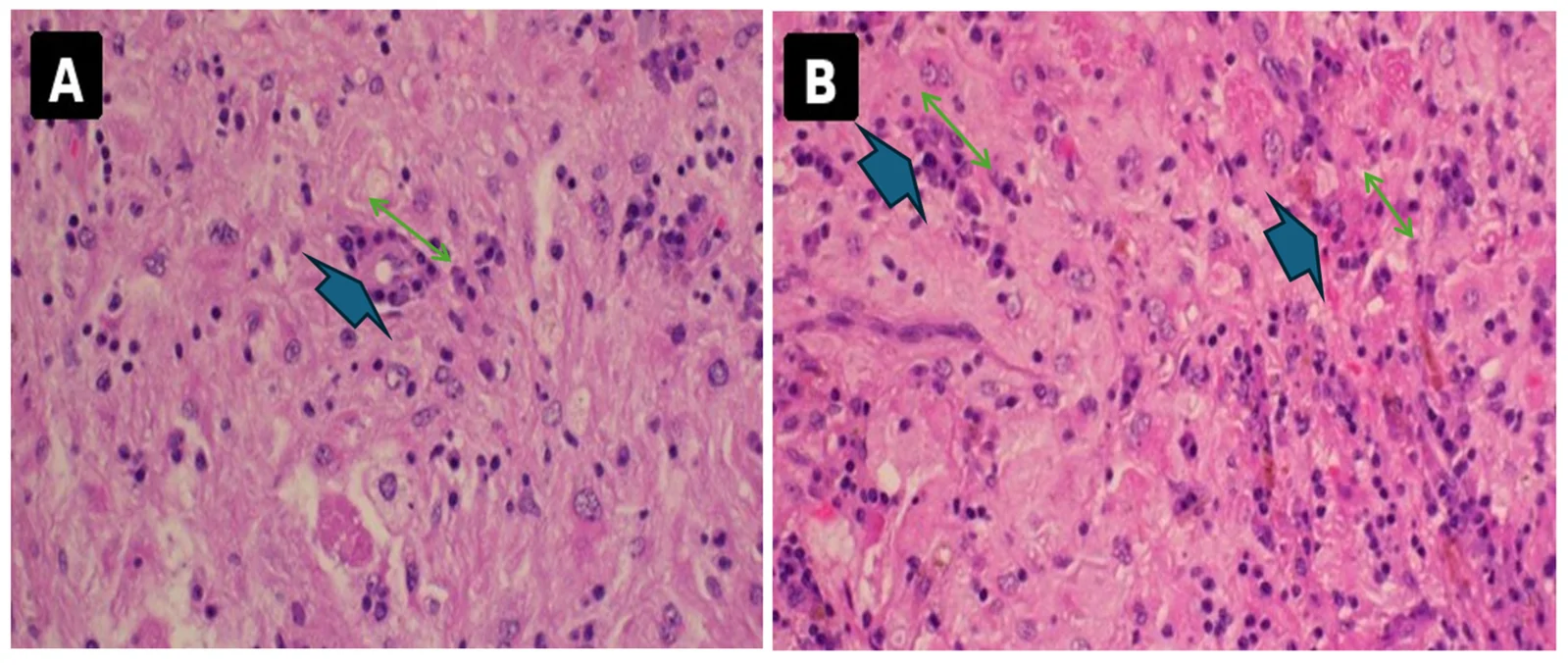
Emperipolesis in Rosai–Dorfman disease. Representative high-power hematoxylin and eosin (H&E)-stained sections (**A**,**B**) demonstrate large lesional histiocytes containing intact inflammatory cells within their abundant cytoplasm, consistent with emperipolesis (blue arrows). Green lines indicate the extent of the highlighted histological areas. Both images were acquired using a ×40 objective.

Histopathological examination showed large pale histiocytes admixed with lymphocytes and plasma cells in a mildly fibrotic background (Figure 2). The lesional histiocytes had round-to-oval nuclei and abundant eosinophilic cytoplasm. Emperipolesis, characterized by intact inflammatory cells within the cytoplasm of lesional histiocytes, was readily identified (Figure 3). No mitotic figures or necrosis were seen throughout the examined lesion. Ki-67 immunohistochemistry was not performed; therefore, a proliferation index was unavailable.

**Figure 4:**
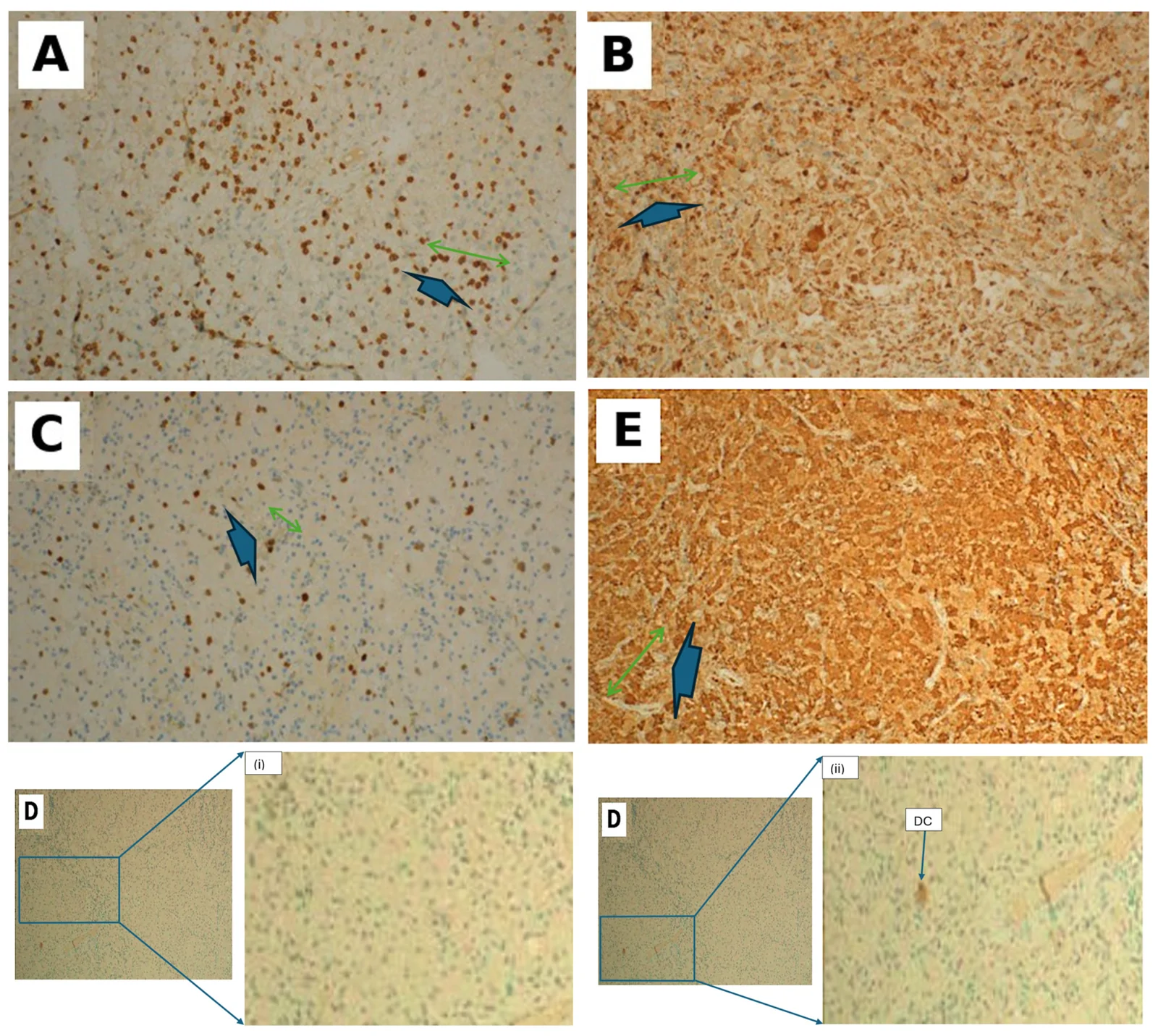
Immunohistochemical profile of the posterior fossa Rosai–Dorfman disease lesion. (**A**) CD3 highlights background T lymphocytes. (**B**) CD68 demonstrates cytoplasmic immunoreactivity in lesional histiocytes. (**C**) Cyclin D1 shows nuclear expression in approximately 30% of lesional histiocytes. (**D**) CD1a immunohistochemistry: (i) lesional histiocytes show no immunoreactivity, whereas (ii) a rare CD1a-positive dendritic cell provides an internal positive control. (**E**) S100 demonstrates diffuse nuclear and cytoplasmic immunoreactivity in lesional histiocytes. Blue arrows indicate representative immunoreactive cells or lesional areas in panels (**A**–**C**,**E**). Blue arrows indicate representative immunoreactive cells or lesional areas in panels (**A**–**C**,**E**), whereas green lines indicate the extent of the highlighted histological areas. Original objective magnifications: ×20 (**A**–**C**,**E**) and ×40 (**D**[i,ii]). CD163 immunohistochemistry was not performed because the reagent was unavailable.

Immunohistochemical analysis demonstrated cytoplasmic CD68 expression and diffuse nuclear and cytoplasmic S100 expression in the lesional histiocytes. Approximately 30% of lesional histiocytes showed nuclear Cyclin D1 staining. No CD1a immunoreactivity was identified in the lesional histiocytes or background cells. CD3 highlighted the background T lymphocytes (Figure 4). Cyclin D1 staining was interpreted as an ancillary, nonspecific finding and was not used independently to establish the diagnosis. Appropriate external controls showed satisfactory reactivity for all immunohistochemical stains. Collectively, the histomorphological and immunohistochemical findings confirmed the diagnosis of intracranial extranodal Rosai–Dorfman disease.

**Figure 5:**
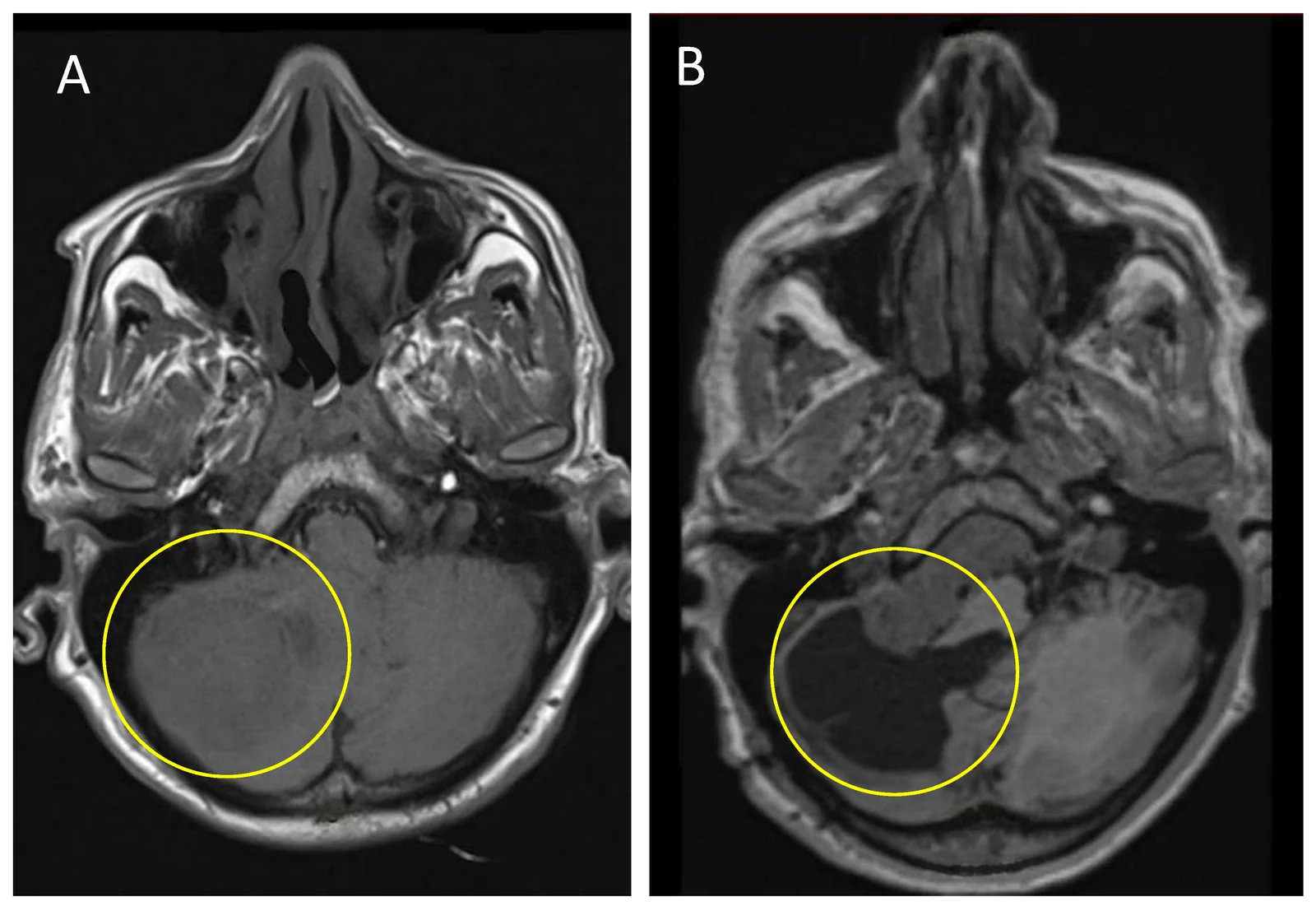
Preoperative and long-term postoperative brain MRI. Comparable axial T1-weighted images obtained preoperatively (**A**) and approximately 2.5 years after surgical resection (**B**). The yellow circle in (**A**) identifies the right posterior fossa mass, whereas the yellow circle in (**B**) indicates the corresponding resection site, showing absence of a visible recurrent mass and resolution of the associated mass effect. Intravenous contrast was not administered during the postoperative examination because the patient declined contrast administration; therefore, assessment for subtle enhancing recurrence was limited.

Postoperatively, the patient experienced improvement in headache, dizziness, and cerebellar manifestations. As part of the planned 6–12-month postoperative surveillance, the patient attended follow-up at seven months, when brain MRI demonstrated complete resection of the right posterior fossa lesion, resolution of cerebellar mass effect, restoration of fourth-ventricular patency, resolution of hydrocephalus, and no recurrence at the resection site (Figure 5). Because gross-total resection was achieved and postoperative imaging showed no residual or recurrent disease, no local adjuvant radiotherapy was administered; the intracranial site was managed with clinical and radiological surveillance. At the one-year clinical assessment, mild cerebellar ataxia persisted; however, the patient remained functionally independent and could walk without assistance.

The patient continued hematological follow-up for progressive systemic RDD. The subsequent lenalidomide–dexamethasone regimen was directed at progressive systemic/extracranial RDD and was not administered as adjuvant treatment for the resected intracranial lesion. Subsequent extracranial progression occurred during postoperative follow-up. Approximately six months after surgery, oral lenalidomide was initiated at 10 mg once daily for 21 days, with concomitant dexamethasone. Only one cycle was completed because the patient did not continue the planned subsequent cycles. The postoperative dexamethasone dose and schedule were unavailable in the verified records. No major treatment-related adverse effects were documented, and treatment response could not be established because the planned course was not completed. PET performed approximately six months after lenalidomide initiation—approximately 12 months after surgery—demonstrated progression of extracranial disease without recurrence at the resected intracranial site. At approximately 2.5 years after surgery, the patient remained neurologically stable, with no visible local intracranial recurrence on non-contrast MRI. Assessment for subtle enhancing recurrence was limited because the patient declined intravenous contrast administration.

A summary of the clinical course, investigations, treatments, and outcomes is provided in Supplementary Table S2.

## 3. Discussion

Intracranial Rosai–Dorfman disease (RDD) is uncommon and most frequently presents as dural-based disease, either in isolation or as part of multisystem involvement [1,3,5,6]. Posterior fossa lesions are particularly important because the confined anatomical space increases the risk of cerebellar or brainstem compression, fourth-ventricular obstruction, and hydrocephalus. In this patient, headache, dizziness, gait ataxia, and right upper limb dysmetria corresponded to compression of the cerebellum and fourth ventricle. The case illustrates how RDD, a histiocytic disorder with heterogeneous clinical behavior, may cause substantial neurological morbidity when it involves the posterior fossa.

The chest-wall and posterior fossa lesions were independently confirmed histopathologically as RDD, supporting multifocal systemic extranodal involvement by the same disease rather than metastasis in the conventional malignant sense. However, because the remaining subcutaneous nodules were not biopsied, their relationship to RDD could not be confirmed.

Central nervous system involvement occurs in approximately 5% of patients with RDD, and approximately 75% of CNS cases involve an intracranial site [1,5]. A recent systematic review and individual patient data meta-analysis identified 327 adults with intracranial RDD across 186 studies, highlighting both the rarity of the condition and the predominantly case-based nature of the evidence [5]. Reported lesion locations were most commonly supratentorial, including intra-axial (52.9%) and extra-axial (20.5%) sites. Sellar or parasellar involvement was reported in 20.8% of patients, whereas infratentorial involvement occurred in 18.0%. Because some patients had lesions at multiple sites, these anatomical categories were not necessarily mutually exclusive. Hydrocephalus was uncommon, occurring in 2.1% of patients. Surgical intervention was performed in 93.6% of cases, most commonly by craniotomy (76.1%), and gross-total resection was achieved in 45.3%. Adjuvant treatments included corticosteroids in 25.1%, radiotherapy in 13.5%, and chemotherapy in 6.7% of patients [5]. These findings indicate that surgery remains the principal diagnostic and therapeutic intervention for accessible symptomatic intracranial lesions, whereas corticosteroids, radiotherapy, and other systemic treatments are generally considered for residual, recurrent, multifocal, or surgically inaccessible disease.

### 3.1. Radiological Features and Differential Diagnosis

Intracranial RDD typically appears on magnetic resonance imaging as a well-defined, strongly enhancing, extra-axial dural-based mass [3,4,7]. Associated findings may include a dural tail, perilesional edema, and substantial mass effect. These features closely resemble those of meningioma and are not sufficiently specific to establish the diagnosis [3,8].

In the present case, the lesion demonstrated strong enhancement, dural attachment, surrounding vasogenic edema, cerebellar compression, and fourth-ventricular effacement. A smaller anterior dural-based enhancing lesion was also present, raising the possibility of multifocal intracranial involvement. Although the patient had biopsy-confirmed systemic RDD, the imaging findings alone could not exclude meningioma, metastatic disease, lymphoma, or another inflammatory lesion.

The differential diagnosis of an enhancing dural-based mass includes meningioma, solitary fibrous tumor, dural metastasis, lymphoma, inflammatory pseudotumor, granulomatous pachymeningitis, Langerhans cell histiocytosis (LCH), and Erdheim–Chester disease (ECD). Dural lymphoma, in particular, may closely resemble a tentorial meningioma [9]. Comparative imaging studies have reported that both CNS RDD and ECD may manifest as multiple masses with meningeal involvement and perilesional edema, whereas CNS LCH more commonly affects the hypothalamic–pituitary axis. Vascular involvement is more characteristic of ECD [7]. Nevertheless, multifocality and known systemic RDD may only raise suspicion for intracranial involvement and are not diagnostic. Because these dural-based entities have substantially overlapping imaging appearances, no individual radiological feature reliably establishes RDD, and histopathological confirmation remains necessary [3,4,7,8,9].

### 3.2. Histopathological and Molecular Diagnosis

Definitive diagnosis of RDD depends on histopathological and immunohistochemical evaluation. Its characteristic morphological feature is emperipolesis, defined as the presence of intact lymphocytes, plasma cells, or other inflammatory cells within the cytoplasm of lesional histiocytes [1,2]. Unlike phagocytosis, emperipolesis does not result in the destruction of the internalized cells. However, it may be focal or inconspicuous and should be interpreted in conjunction with the overall histological appearance and immunophenotype.

RDD histiocytes typically express S100, CD68, and CD163 but lack CD1a expression [1,2,6]. In the present case, the lesion showed large pale histiocytes admixed with lymphocytes and plasma cells in a mildly fibrotic background. Emperipolesis, characterized by intact inflammatory cells within the cytoplasm of lesional histiocytes, was identified. The extent of emperipolesis was not formally quantified and is therefore reported qualitatively rather than as a numerical frequency. No mitotic figures or necrosis were seen. The lesional histiocytes demonstrated cytoplasmic CD68 expression and diffuse nuclear and cytoplasmic S100 expression, whereas no CD1a immunoreactivity was identified in the lesional histiocytes or background cells. Approximately 30% of lesional histiocytes showed nuclear Cyclin D1 expression; however, this was interpreted as an ancillary, nonspecific finding and was not used independently to establish the diagnosis [10]. This combination of morphology and immunophenotype supported RDD and helped distinguish it from Langerhans cell histiocytosis, in which lesional cells typically express CD1a and langerin, and Erdheim–Chester disease, in which histiocytes are generally negative or only weakly positive for S100 [1,2,6]. CD163 immunohistochemistry was not performed because the stain was unavailable, and Ki-67 immunohistochemistry was not performed; therefore, a proliferation index was unavailable.

The principal histopathological and immunohistochemical features distinguishing RDD from meningioma, Langerhans cell histiocytosis, and Erdheim–Chester disease are summarized in Table 1 [1,2,6,11,12].

Other pathological mimics of a dural-based mass include meningioma, solitary fibrous tumor, dural lymphoma, and metastatic carcinoma. Meningioma demonstrates meningothelial differentiation rather than histiocytic proliferation with emperipolesis; solitary fibrous tumor shows spindle-cell proliferation; dural lymphoma consists of a neoplastic lymphoid population; and metastatic carcinoma retains epithelial differentiation [11]. These entities lack the characteristic combination of pale histiocytes with emperipolesis and an S100-positive, CD68-positive, and CD1a-negative immunophenotype observed in the present case. Integration of the morphological, immunohistochemical, and radiological findings therefore supported the diagnosis of RDD.

#### Bottom of Form

The previously biopsied chest-wall lesion was negative for the BRAF V600E mutation. Although this alteration is more commonly associated with LCH and ECD, its absence does not exclude RDD. Somatic alterations involving the mitogen-activated protein kinase (MAPK) pathway have been identified in a subset of patients with RDD and may have therapeutic relevance in severe, refractory, or unresectable disease [6,12]. Molecular findings must therefore be interpreted within the complete clinical, morphological, and immunohistochemical context.

### 3.3. Management and Clinical Outcome

Because CNS RDD is rare, its management is based mainly on consensus recommendations, retrospective studies, and case reports rather than randomized comparative trials [1,5,6,13]. Treatment should be individualized according to the lesion’s location, neurological manifestations, resectability, and systemic disease burden. Observation may be appropriate for asymptomatic or stable lesions, whereas corticosteroids, immunomodulatory agents, chemotherapy, radiotherapy, or targeted therapy may be considered for residual, recurrent, disseminated, or surgically inaccessible disease.

Surgery has both diagnostic and therapeutic roles in patients with symptomatic intracranial RDD, particularly when lesions cause neurological deficits, substantial mass effect, or obstructive hydrocephalus [1,5,8,13]. In this patient, headache and cerebellar dysfunction progressed despite corticosteroid therapy, while imaging demonstrated cerebellar compression, fourth-ventricular effacement, and mild hydrocephalus. Gross-total resection provided definitive tissue diagnosis, relieved the posterior fossa mass effect, and restored fourth-ventricular patency. The patient experienced sustained improvement in headache, dizziness, and cerebellar manifestations. Mild ataxia persisted, but he remained independently ambulatory. Follow-up MRI demonstrated complete resection without recurrent hydrocephalus or local tumor recurrence. Accordingly, no local adjuvant treatment was administered after gross-total resection. This approach is consistent with the individualized management of intracranial RDD, in which corticosteroids, radiotherapy, or systemic therapy are generally considered for residual, recurrent, multifocal, or surgically inaccessible disease [1,5,6,13].

Local intracranial control does not necessarily indicate control of multisystem RDD. Despite the absence of recurrence at the resected site, follow-up positron emission tomography demonstrated progression of the patient’s extracranial disease. This difference highlights the need to evaluate local neurological outcomes separately from systemic disease activity. Continued hematological surveillance and individualized systemic treatment are therefore required following successful neurosurgical management [1,6,13]. The patient received lenalidomide and dexamethasone, a regimen that has demonstrated activity in a prospective phase 2 study of RDD, although treatment responses remain variable [14].

MAPK pathway-directed therapies, including mitogen-activated protein kinase (MEK) inhibitors, may be considered in selected patients with actionable alterations and refractory, disseminated, or unresectable disease [6,12]. Nevertheless, systemic therapy does not replace urgent surgical decompression when a posterior fossa lesion causes progressive neurological dysfunction or threatens cerebrospinal fluid pathways.

### 3.4. Perioperative and Multidisciplinary Considerations

Perioperative management was complicated by anemia and serological incompatibility during preoperative crossmatching. Extended testing did not identify a fully compatible donor unit. After consultation with the transfusion medicine service, a least-incompatible, irradiated, leukocyte-reduced packed red blood cell unit was selected and administered under close monitoring without an acute transfusion reaction.

The lesion was highly vascular, and the estimated intraoperative blood loss was approximately 500 mL. One packed red blood cell unit was transfused intraoperatively without complication. Although transfusion incompatibility is not a characteristic feature of RDD, it substantially affected the timing and planning of surgery. Collaboration among neurosurgery, hematology, anesthesiology, pathology, radiology, and transfusion medicine was essential to achieving safe resection and a favorable neurological outcome.

### 3.5. Clinical Relevance and Limitations

Previous Saudi Arabian reports have described RDD involving intracranial, orbital, infratemporal, and spinal sites [15,16]. To the best of our knowledge, the present case is the first reported Saudi case of posterior fossa RDD producing cerebellar compression, fourth-ventricular obstruction, and hydrocephalus requiring neurosurgical resection. Its principal contribution is the radiological–pathological correlation of a rare meningioma-like posterior fossa lesion and the demonstration that sustained local intracranial control may coexist with progressive systemic disease.

This report has limitations inherent to a single-patient observation, including limited generalizability and the potential for retrospective selection and information bias. It cannot establish causality, comparative treatment effectiveness, or broadly generalizable management recommendations. The smaller anterior dural-based lesion was not biopsied, preventing pathological confirmation of multifocal intracranial RDD. In addition, the remaining subcutaneous nodules were not biopsied; therefore, their classification as RDD lesions or neurofibromas could not be histopathologically confirmed. Molecular testing beyond BRAF V600E was unavailable, and longer radiological follow-up would be valuable. An additional pathological limitation was that CD163 immunohistochemistry was not performed because the stain was unavailable. Nevertheless, the diagnosis was supported by the characteristic morphology, including emperipolesis, together with the available S100-positive, CD68-positive, and CD1a-negative immunophenotype. Furthermore, the literature on CNS RDD consists predominantly of retrospective studies and case reports with heterogeneous interventions and follow-up periods [5,8]. Conclusions regarding optimal treatment must therefore remain cautious.

Nevertheless, this case reinforces three clinically relevant points. First, RDD should be considered in the differential diagnosis of a meningioma-like dural mass, particularly in a patient with known systemic disease. Second, definitive diagnosis requires histopathological and immunohistochemical confirmation because imaging findings are nonspecific. Third, timely surgical decompression can provide diagnostic confirmation and sustained neurological benefit in symptomatic posterior fossa disease, but continued systemic surveillance remains necessary.

## 4. Conclusions

Intracranial RDD should be considered in the differential diagnosis of strongly enhancing, dural-based posterior fossa masses, particularly in patients with known systemic disease. Because its radiological appearance may closely resemble that of a meningioma, definitive diagnosis requires histopathological and immunohistochemical confirmation. In symptomatic posterior fossa disease causing neurological deficits, mass effect, or hydrocephalus, timely surgical resection can provide diagnostic confirmation, effective decompression, and sustained neurological improvement. Continued multidisciplinary surveillance remains necessary because successful local intracranial control does not necessarily indicate control of systemic RDD.

## Figures and Tables

**Table 1:** Concise differential diagnosis of Rosai–Dorfman disease and selected mimics of an intracranial dural-based lesion.

Entity	S100	CD68/CD163	CD1a/Langerin	Other Diagnostically Useful IHC	Characteristic Features
Rosai–Dorfman disease (RDD)	Positive; typically nuclear and cytoplasmic	Positive	Negative	Cyclin D1 may be expressed in a subset of lesional histiocytes; it is an ancillary, nonspecific finding and is not independently diagnostic.	Large pale histiocytes in a mixed inflammatory background; emperipolesis is characteristic; fibrosis may be prominent in extranodal lesions.
Meningioma	Usually negative; variable or focal expression may occur depending on subtype	Negative in neoplastic cells; background macrophages may be positive	Negative	EMA and SSTR2A are commonly positive and support meningothelial differentiation.	Meningothelial cells arranged in lobules or whorls; psammoma bodies may be present.
Langerhans cell histiocytosis (LCH)	Positive	CD68 often positive; CD163 variable	Positive	—	Histiocytes with grooved or folded nuclei, often in an eosinophil-rich inflammatory background; Langerin positivity is characteristic; Birbeck granules may be demonstrated ultrastructurally.
Erdheim–Chester disease (ECD)	Usually negative; weak or focal staining may occur	Positive	Negative	—	Foamy or lipid-laden histiocytes, Touton giant cells, and fibrosis; characteristic systemic findings include symmetric long-bone osteosclerosis and perinephric or periaortic infiltration.
IgG4-related disease (IgG4-RD)	Usually negative in the lesional process	Highlights background histiocytes rather than the lesional plasma-cell–rich process	Negative	Increased IgG4-positive plasma cells and an elevated IgG4/IgG ratio support the diagnosis when interpreted with morphology and clinical findings.	Dense lymphoplasmacytic inflammation with storiform fibrosis and, in some sites, obliterative phlebitis; may present as hypertrophic pachymeningitis or a dural-based mass.
Dural lymphoma	Usually negative in neoplastic lymphoid cells	Negative in neoplastic cells; background macrophages may be positive	Negative	Lineage markers (e.g., CD20/PAX5 for B-cell lesions) and demonstration of clonality or light-chain restriction support the diagnosis.	Monomorphic neoplastic lymphoid infiltrate; plasma-cell differentiation may occur and can mimic a plasma-cell–rich inflammatory lesion.
Plasma cell granuloma (inflammatory pseudotumor)	Variable; not diagnostic	Highlights admixed histiocytes	Negative	Plasma cells are typically polytypic for kappa and lambda light chains; immunostains should exclude lymphoma and IgG4-RD.	Polyclonal plasma-cell–rich inflammatory infiltrate with variable fibrosis and spindle-cell proliferation; diagnosis requires exclusion of infection, IgG4-RD, and hematolymphoid neoplasia.

Note: S100, CD68, CD163, CD1a, Langerin, Cyclin D1, EMA, SSTR2A, and IgG4 are immunohistochemical markers rather than molecular markers. Immunophenotypic findings should be interpreted together with morphology, clinical presentation, imaging, and, when indicated, molecular or clonality testing. Cyclin D1 expression may occur in RDD but is ancillary and nonspecific. MAPK-pathway alterations may occur in RDD, LCH, and ECD and are not independently diagnostic of any one entity [1,2,6,10,11,12]. Abbreviations: CD, cluster of differentiation; ECD, Erdheim–Chester disease; EMA, epithelial membrane antigen; IgG4, immunoglobulin G4; IgG4-RD, IgG4-related disease; IHC, immunohistochemistry; LCH, Langerhans cell histiocytosis; MAPK, mitogen-activated protein kinase; PAX5, paired box 5; RDD, Rosai–Dorfman disease; SSTR2A, somatostatin receptor 2A.

## Data Availability

The data supporting this case report are contained within the article. Additional patient-level data are not publicly available because of privacy and confidentiality restrictions. Further inquiries may be directed to the corresponding author, subject to patient consent and institutional approval.

## Supplementary Materials

The following supporting information can be downloaded at: https://www.mdpi.com/article/10.3390/diagnostics16203384/s1: Table S1: CARE Checklist; Table S2: Clinical Timeline.

## Abbreviations

The following abbreviations are used in this manuscript:

Abbreviation	Definition
CARE	Case REport
CNS	Central nervous system
CT	Computed tomography
ECD	Erdheim–Chester disease
H&E	Hematoxylin and eosin
LCH	Langerhans cell histiocytosis
MAPK	Mitogen-activated protein kinase
MEK	Mitogen-activated protein kinase kinase
MRI	Magnetic resonance imaging
PET	Positron emission tomography
RDD	Rosai–Dorfman disease
