# Supplementary material for: Intracranial Rosai–Dorfman Disease Presenting as a Posterior Fossa Dural-Based Mass Mimicking Meningioma: A Case Report
Source: Diagnostics (Basel). 2026 Oct 18;16(20):3384. doi: 10.3390/diagnostics16203384 (PMC13702947; doi:10.3390/diagnostics16203384)
Supplement: Supplementary file 1 [file diagnostics-16-03384-s001.zip › diagnostics-4569016-supplementary.pdf]

Supplementary Table S1. CARE Checklist

Intracranial Rosai–Dorfman Disease Presenting as a Posterior Fossa Dural-Based Mass Mimicking Meningioma: A Case Report

This checklist maps the CARE reporting items to the revised manuscript. Section references should be updated if pagination changes during typesetting.

Topic	Item	CARE reporting requirement	Location in revised manuscript
Title	1	The diagnosis or intervention of primary focus followed by “case report”.	Title.
Keywords	2	Two to five keywords that identify the diagnosis or intervention, including “case report”.	Keywords: Rosai–Dorfman disease; non-Langerhans cell histiocytosis; intracranial lesion; posterior fossa; dural-based mass; meningioma mimic; emperipolesis; case report.
Abstract	3a	Introduction—What is unique about this case and what does it add to the literature?	Abstract—Background and Clinical Significance.
Abstract	3b	Main symptoms and/or important clinical findings.	Abstract—Case Presentation.
Abstract	3c	Main diagnoses, therapeutic interventions, and outcomes.	Abstract—Case Presentation and Conclusions.
Introduction	4	Brief background summary explaining why the case is unique, with relevant literature.	Section 1, Introduction.
Patient information	5a	De-identified demographic and other patient-specific information.	Section 2, Case Presentation: 58-year-old man and relevant comorbidities.
Patient information	5b	Main concerns and symptoms of the patient.	Section 2, Case Presentation: headache, nausea, severe dizziness, gait instability, cerebellar ataxia, and right upper-limb dysmetria.
Patient information	5c	Medical, family, and psychosocial history, including relevant genetic information.	Section 2, Case Presentation: diabetes mellitus, solitary kidney, anemia, gastrointestinal bleeding, systemic extranodal RDD, weight loss, and BRAF V600E result. Family and psychosocial history was unavailable or not relevant.
Patient information	5d	Relevant past interventions and their outcomes.	Section 2, Case Presentation: prednisone 20 mg once daily for two months produced limited radiological response and hyperglycemia; it was replaced with dexamethasone 4 mg once daily for four months. Perioperative tapering and hydrocortisone coverage are described.
Clinical findings	6	Relevant physical examination and other significant clinical findings.	Section 2, Case Presentation: conscious, oriented, and hemodynamically stable; cerebellar ataxia and right upper-limb dysmetria; preserved cranial nerve, motor, and sensory findings.
Timeline	7	Important information from the patient’s history organized as a timeline.	Supplementary Table S2, Clinical Timeline.
Diagnostic assessment	8a	Diagnostic methods, including examination, laboratory testing, imaging, and histopathology.	Section 2, Case Presentation; Figures 1–4. Assessment included neurological examination, CT, MRI (including enlarged lateral ventricles in Figure 1D), histopathology, and immunohistochemistry.
Diagnostic assessment	8b	Diagnostic challenges, including access, financial, or cultural issues.	Section 2, Case Presentation: radiological overlap with meningioma, metastatic disease, and inflammatory lesions; complex transfusion incompatibility. No financial or cultural barriers were reported.

Topic	Item	CARE reporting requirement	Location in revised manuscript
Diagnostic assessment	8c	Diagnosis, including differential diagnoses considered.	Sections 2 and 4.1–4.2 and Table 1: intracranial extranodal RDD; relevant differentials include meningioma, solitary fibrous tumor, dural metastasis, lymphoma, inflammatory or granulomatous pachymeningeal disease, LCH, and ECD.
Diagnostic assessment	8d	Prognostic characteristics, such as staging, where applicable.	Section 2: systemic extranodal RDD with symptomatic posterior fossa involvement, edema, fourth-ventricular effacement, and mild obstructive hydrocephalus. Formal staging was not applicable.
Therapeutic intervention	9a	Types of therapeutic intervention, including pharmacologic, surgical, preventive, or self-care treatment.	Section 2: prednisone and dexamethasone; microsurgical resection through a suboccipital approach with gross-total piecemeal excision of the tumor and involved dura; perioperative transfusion; subsequent lenalidomide–dexamethasone for systemic disease. No preoperative EVD, ETV, or other CSF diversion was performed.
Therapeutic intervention	9b	Administration details, including dose, strength, duration, and frequency, where available.	Section 2 and Supplementary Table S2: prednisone 20 mg orally once daily for two months; dexamethasone 4 mg orally once daily for four months, followed by perioperative tapering with hydrocortisone coverage; lenalidomide 10 mg orally once daily for 21 days (one cycle). The postoperative dexamethasone dose and schedule were unavailable in the verified records.
Therapeutic intervention	9c	Changes in intervention, with rationale.	Section 2: prednisone was replaced after limited radiological response and steroid-associated hyperglycemia; surgery was undertaken for progressive cerebellar manifestations, mass effect, fourth-ventricular compression, mild hydrocephalus, and definitive diagnosis. Later systemic treatment was directed at progressive extracranial RDD.
Follow-up and outcomes	10a	Clinician- and patient-assessed outcomes, when appropriate.	Section 2: postoperative improvement in headache, dizziness, and cerebellar manifestations; mild residual ataxia at one year with independent ambulation; neurological stability at approximately two years.
Follow-up and outcomes	10b	Important follow-up diagnostic and other test results.	Section 2; Figure 5: MRI at seven months showed complete resection, restored fourth-ventricular patency, resolved mass effect and hydrocephalus, and no local recurrence; one-year clinical assessment; PET six months after systemic treatment initiation; latest documented follow-up at approximately two years.
Follow-up and outcomes	10c	Intervention adherence and tolerability, and how these were assessed.	Section 2: transfusions were tolerated without acute reaction. Only one 21-day lenalidomide cycle was completed because the patient did not adhere to subsequent planned cycles; response could not be established. No major treatment-related adverse effects were documented.
Follow-up and outcomes	10d	Adverse and unanticipated events.	Section 2: prednisone-associated hyperglycemia was treated and monitored; no acute transfusion reaction or intraoperative complication was documented; mild residual cerebellar ataxia persisted.

Topic	Item	CARE reporting requirement	Location in revised manuscript
Discussion	11a	Strengths and limitations of the approach to the case.	Section 4.5, Clinical Relevance and Limitations: limited generalizability, potential retrospective selection and information bias, inability to establish causality or comparative effectiveness, unbiopsied lesions, unavailable additional molecular testing, absent CD163 testing, and limited follow-up.
Discussion	11b	Relevant medical literature.	Sections 4.1–4.5, Table 1, and References, including the literature overview of 327 adult intracranial RDD cases across 186 studies.
Discussion	11c	Rationale for conclusions, including assessment of possible causes.	Sections 4.2–4.5 and Section 5, Conclusions.
Discussion	11d	Primary take-away lessons from the case report.	End of Section 4.5 and Section 5, Conclusions.
Patient perspective	12	The patient’s perspective or experience, when appropriate.	Not available; no patient-perspective narrative was obtained.
Informed consent	13	Confirmation that informed consent was obtained.	Informed Consent Statement: written consent was obtained for publication of the clinical details and accompanying radiological and histopathological images.

Supplementary Table S2. Clinical Timeline

Time/phase	Clinical events, investigations, treatment, and outcome
**Initial clinical evaluation**	The patient was evaluated for iron-deficiency anemia and gastrointestinal bleeding. Upper gastrointestinal endoscopy showed gastritis. Multiple subcutaneous nodules were observed and were initially suspected clinically to represent neurofibromatosis.
**Diagnostic confirmation of systemic disease**	A representative chest-wall nodule was biopsied and showed extranodal Rosai–Dorfman disease. The remaining subcutaneous nodules were not biopsied. Molecular testing of the chest-wall lesion showed no detectable BRAF V600E mutation.
**Before neurosurgical referral**	A posterior fossa mass had been identified during evaluation for headache, but the patient initially declined surgery. For systemic RDD, oral prednisone 20 mg once daily was administered for two months and produced a limited radiological response with hyperglycemia. Prednisone was then replaced with oral dexamethasone 4 mg once daily, which was continued for four months, from approximately two months after diagnosis until surgery. The disease remained radiologically stable during this preoperative dexamethasone period.
**Neurosurgical presentation**	The patient developed worsening headache, nausea, severe dizziness, gait instability, cerebellar ataxia, and right upper-limb dysmetria. CT and MRI showed a strongly enhancing right posterior fossa dural-based mass with vasogenic edema, cerebellar compression, fourth-ventricular effacement, and mild obstructive hydrocephalus; a smaller anterior dural-based enhancing lesion was also present.
**Preoperative period**	Multidisciplinary assessment was undertaken. Anemia and serological incompatibility complicated crossmatching. A least-incompatible, irradiated, leukoreduced O-positive packed red blood cell unit was transfused under monitoring without an acute reaction.
**Surgery**	Posterior fossa craniotomy and gross-total resection were performed. The highly vascular lesion was associated with approximately 500 mL estimated blood loss; one packed red blood cell unit was transfused without complication.
**Pathological diagnosis**	Histopathological examination demonstrated pale histiocytes in a mixed inflammatory background with emperipolesis. Lesional histiocytes expressed S100 and CD68, approximately 30% showed nuclear Cyclin D1 staining, and CD1a was negative, supporting intracranial extranodal RDD.
**Early postoperative course**	Headache, dizziness, and cerebellar manifestations improved after surgery.
**Seven months after surgery**	Follow-up MRI demonstrated complete resection of the posterior fossa lesion, resolution of mass effect, restoration of fourth-ventricular patency, and resolution of hydrocephalus.
**One year after surgery**	Mild cerebellar ataxia persisted, but the patient remained functionally independent and walked without assistance.
**Systemic follow-up**	Subsequent extracranial progression occurred during postoperative follow-up. Approximately six months after surgery, oral lenalidomide was initiated at 10 mg once daily for 21 days, with concomitant dexamethasone. Only one cycle was completed because the patient did not continue the planned subsequent cycles; the postoperative dexamethasone dose and schedule were unavailable in the verified records. PET performed approximately six months after lenalidomide initiation—approximately 12 months after surgery—demonstrated progression of extracranial disease without recurrence at the resected intracranial site.
Approximately 2.5 years after surgery	At approximately 2.5 years after surgery, the patient remained neurologically stable. Non-contrast brain MRI showed no visible local intracranial recurrence and resolution of the associated mass effect. Assessment for subtle enhancing recurrence was limited because the patient declined intravenous contrast administration.
